# The Ragulator complex: delving its multifunctional impact on metabolism and beyond

**DOI:** 10.1186/s41232-023-00278-2

**Published:** 2023-05-12

**Authors:** Kohei Tsujimoto, Hyota Takamatsu, Atsushi Kumanogoh

**Affiliations:** 1 Department of Respiratory Medicine and Clinical Immunology, Osaka University Graduate School of Medicine, Suita, Osaka Japan; 2 Department of Immunopathology, Immunology Frontier Research Center (IFReC), Osaka University, Suita, Osaka Japan; 3 Center for Infectious Diseases Education and Research (CiDER), Osaka University, Suita, Osaka Japan; 4 Integrated Frontier Research for Medical Science Division, Institute for Open and Transdisciplinary Research Initiatives (OTRI), Osaka University, Suita, Osaka Japan; 5 Japan Agency for Medical Research and Development — Core Research for Evolutional Science and Technology (AMED–CREST), Osaka University, Osaka, Japan; 6 Center for Advanced Modalities and DDS (CAMaD), Osaka University, Osaka, Japan

**Keywords:** Lysosomes, The Ragulator complex, Inflammation, LAMTOR, mTORC1

## Abstract

Our understanding of lysosomes has undergone a significant transformation in recent years, from the view that they are static organelles primarily responsible for the disposal and recycling of cellular waste to their recognition as highly dynamic structures. Current research posits that lysosomes function as a signaling hub that integrates both extracellular and intracellular stimuli, thereby regulating cellular homeostasis. The dysregulation of lysosomal function has been linked to a wide range of diseases. Of note, lysosomes contribute to the activation of mammalian target of rapamycin complex 1 (mTORC1), a key regulator of cellular metabolism. The Ragulator complex, a protein complex anchored on the lysosomal membrane, was initially shown to tether the mTORC1 complex to lysosomes. Recent research has substantially expanded our understanding of the roles of the Ragulator complex in lysosomes, including roles in the regulation of metabolism, inflammation, cell death, cell migration, and the maintenance of homeostasis, via interactions with various proteins. This review summarizes our current knowledge on the diverse functions of the Ragulator complex, highlighting important protein interactions.

## Background

Lysosomes are membrane-enclosed cytoplasmic organelles responsible for the degradation of a variety of biological macromolecules. Lysosomes maintain the acidic environment via lysosomal v-ATPase, providing appropriate conditions for the functions of more than approximately 60 proteases, lipases, nucleases, and other hydrolytic enzymes [1–3]. The acidic environment is suitable for the breakdown of major macromolecules, including proteins, lipids, carbohydrates, and nucleic acids [4]. Owing to this feature, it was believed for many years that the role of the lysosome is limited to degradation. However, our view of the lysosome has evolved from a simple, degradative center to a dynamic signaling hub capable of detecting and interpreting cellular signals to regulate downstream responses [2, 5]. For example, recent studies have shown that lysosomes are involved in a variety of tasks related to metabolic adaptation, nutrient sensing, inflammation, cell differentiation, membrane repair, and the quality control of proteins and organelles [3, 5–7]. Abnormalities in lysosomal function result in a broad range of diseases [3, 5]. For example, many autoimmune diseases are associated with autophagy and changes in lysosomal biogenesis, acidification, and cathepsin activity [8–10].

A major factor contributing to the pleiotropic roles of lysosomes is their connection with mechanistic target of rapamycin complex 1 (mTORC1), a master regulator of cellular metabolism. mTOR is an evolutionarily conserved phosphatidylinositol-3-kinase (PI3K)-like serine/threonine kinase integrating nutrient and energy cues to balance catabolic and anabolic processes [11, 12]. In addition to metabolism, mTORC1 regulates diverse cellular processes (e.g., cell proliferation, cell death, protein synthesis, and autophagy) via multiple signaling pathways [13, 14]. The activity of mTORC1 is spatiotemporally regulated in a highly detailed manner. Under appropriate intracellular and extracellular conditions, mTORC1 is translocated to and is activated on lysosomes.

**Fig. 1:**
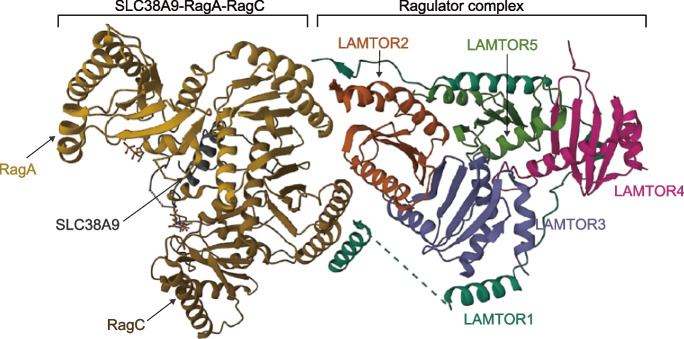
CryoEM structure of the SLC38A9-RagA-RagC-Ragulator complex. Overall structures of the Ragulator complex. Lamtor1 wraps around Lamtor2–5, stabilizing the Ragulator complex, and the LAMTOR2/3 heterodimer associates with RagA/B and RagC/D GTPases. The original image is from the RCSB PDB (rcsb.org), PDB ID: 6WJ2 (from S. A., Lawrence, R. E., Hurley, J. H. (2020). Structural mechanism for amino acid-dependent Rag GTPase nucleotide state switching by SLC38A9. Nat Struct Mol Biol 27: 1017–1023)

The lysosomal Ragulator complex plays a critical role in the mTOR signal transduction pathway by tethering the mTORC1 complex to the lysosomal surface [15–17]. Briefly, the Ragulator complex is a pentamer containing Lamtor1/p18, Lamtor2/p14–Lamtor3/MP1, and Lamtor4/p10–Lamtor5/HBXIP [17]. Lamtor1 wraps around Lamtor2–5, stabilizing the Ragulator complex, and the protein levels of Lamtor2–5 are reduced in cases of Lamtor1 deficiency (Fig. 1) [18]. A similar phenomenon has been reported in case of Lamtor2 deficiency [19]. Lamtor1 is directly responsible for anchoring the complex to the lysosomal membrane by its N-terminal myristoyl and palmitoyl groups [20]. A crystal structure analysis revealed that extensive hydrophobic interactions occur between Lamtor1 and the roadblock domain pairs Lamtor2–Lamtor3 and Lamtor4–Lamtor5, with no substantial contact with Lamtor2–Lamtor3 and Lamtor4–Lamtor5 subcomplexes [21].

In addition to functioning as a lysosomal membrane scaffold for Rag GTPases to recruit and activate mTORC1, the Ragulator complex can regulate many cellular functions by interacting with various proteins. In this review, we summarize recent research on the role of lysosomes in maintaining cellular homeostasis, focusing on the role of the Ragulator complex. We emphasize the concept that the Ragulator complex serves as a platform for integrating various information types by interaction with other proteins. Though many reports have shown a relationship between the Ragulator complex and cancer, we will not focus on this area of research in this review.

### Lysosomes in cellular metabolism

**Fig. 2:**
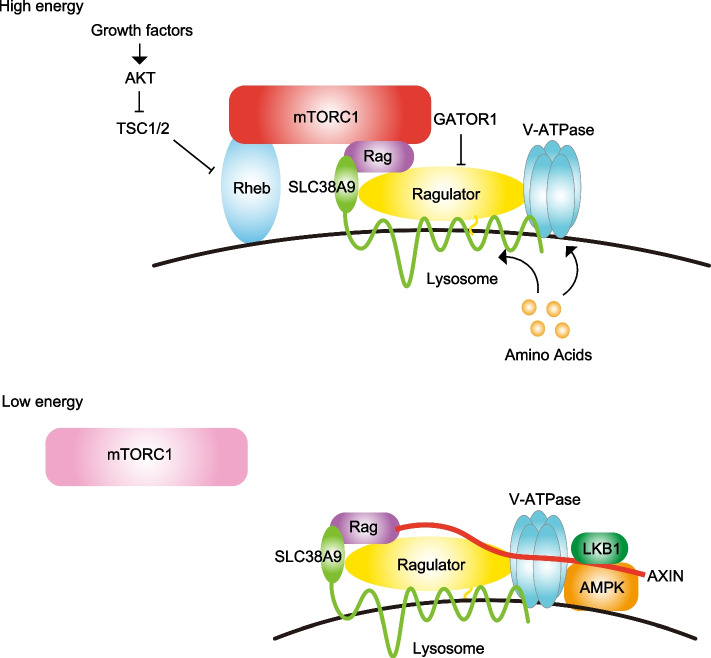
Schematic model of mTORC1 and AMPK regulation by the Ragulator complex. When nutrients are abundant, active v-ATPase stimulates the GEF activity of Ragulator toward Rags and activates mTORC1. Active mTORC1 switches on anabolic pathways. Upon glucose starvation, inactive v-ATPase and the Ragulator complex become accessible for AXIN binding, which in turn inhibits the GEF activity of the Ragulator complex, leading to the dissociation of mTORC1 from lysosomes. Starvation leads to the translocation of AXIN/LKB1 to lysosomes, where LKB1 phosphorylates and activates AMPK, switching on catabolism

After degradation by lysosomes, free fatty acids, monosaccharides, and amino acids are transported to the cytoplasm, where they can be reused in anabolic processes [3]. Lysosomes function as signaling hubs that coordinate the balance between anabolism and catabolism, and this function is closely related to mTORC1. As the master regulator of cellular metabolism, mTORC1 integrates information from hormones, such as insulin, IGF-1, leptin, and adiponectin, to regulate the cellular energy status by sensing the availability of amino acids, glucose, and cholesterol [13, 14, 22]. The activation of mTORC1 depends on the nucleotide-loading state of the Rheb and RAS-related GTP-binding proteins (RAGs) [23, 24] (Fig. 2). Activation of mTORC1 by growth factors is mainly regulated by the activation state of Rheb that localizes to lysosomes [23, 24]. Rheb is activated in GTP-bound state, and its level is controlled by the TSC1-TSC2 complex (tuberous sclerosis protein complex) with GAP (GTPase-activating protein) activity [23, 24]. Since TSC1-TSC2 enhances GTPase activity and converts Rheb-GTP to GDP-bound form, it serves as an inactivator of Rheb. In response to growth factors, including insulin, serine/threonine kinase AKT phosphorylates TSC2 and inhibits the TSC complex, and TSC1-TSC2 dissociates from Rheb on lysosomes [23]. As a result, Rheb becomes GTP-bound and activates mTORC1. On the other hand, the activation of mTORC1 by amino acids is controlled by the nucleotide-loading state of Rag [25, 26]. Mammals have four Rag proteins, RagA to RagD, which form obligate heterodimers comprising RagA or RagB together with RagC or RagD [26]. In the presence of amino acids, the Rag dimers are in an “active” conformation with GTP-bound RagA/GDP-bound RagC complex. While the Rag dimers themselves do not possess the ability to activate mTORC1, the active Rag dimers instead bind to Raptor, a major component of mTORC1, and facilitate the translocation and localization of mTORC1 from the cytoplasm to the lysosome when amino acids, glucose, and other nutrients are readily available. The Rag dimers are not lipid modified and localize to the lysosome by binding to the Ragulator complex [15]. In addition to its role as an anchor, the Ragulator complex acts as a guanine nucleotide exchange factor (GEF), exchanging GDP for GTP on RagA when amino acids are present [27, 28]. Activated mTORC1 interacts with its obligate activator Rheb-GTP to phosphorylate substrates, including S6K1, 4E-BP1, and ULK1, thereby promoting ribosomal biogenesis, translation, and lipogenesis while suppressing autophagy [13, 22]. In contrast, if the environment is not permissive, phosphorylation by AMP-activated protein kinase (AMPK) stabilizes the complex to promote GTPase-activating protein activity and inhibition of mTORC1 signaling [29]. The inactivation of mTOR signaling and the formation of the AMPK complex contribute to the upregulation of catabolic pathways, while autophagy is activated [30]. In this way, mTORC1 activation is regulated by its localization, and the Ragulator complex tethers the mTORC1 complex to the lysosomal surface [15–17].

The Ragulator complex has been evaluated extensively by cryo-electron microscopy and crystallographic analyses [17, 20, 31–35]. These structural analyses have revealed that the Ragulator complex has various roles in the response to cellular conditions via interactions with effector proteins. The Ragulator complex serves as an essential activation platform for metabolic signaling through interactions with extracellular signal-regulated kinase-mitogen-activated protein kinase (ERK–MAPK) and mTOR cascades [15, 36]. LAMTOR2/3 interact with MAPK kinase 1/MEK1, thereby facilitating the activation of MAPK3/ERK1 [37]. The LAMTOR2/3 heterodimer is also associated with RagA/B and RagC/D GTPases, and the Ragulator complex serves as a GEF, pushing Rag GTPases toward the active state [27, 28]. However, the Ragulator complex has a more precise role in mTORC1 activation.

How cells sense their nutrient status, particularly amino acid availability, and transmit this information to mTORC1, much remains unknown [38]. However, the sensors for leucine and arginine are well characterized. Sestrin2, SAR1B, and LRS are reported as cytosolic leucine sensors [39–41]. Sestrin2 and SAR1B control mTORC1 activity in a leucine-dependent manner by interacting with GATOR2 [39, 40]. Other amino acid sensors include lysosomal amino acid sensor SLC38A9 and transmembrane 4 L six family member 5 [42, 43]. Amino acids obtained by catabolism are transported out of lysosomes by SLC38A9 in an arginine-regulated manner [42, 44]. SLC38A9 also transduces amino acids to mTORC1 for the switching between anabolic mTORC1 and catabolic AMPK activities [42, 45]. The Ragulator complex transfers information about amino acids in lysosomes to mTORC1 via interaction with SLC38A9. The Ragulator complex also interacts with the vacuolar v-ATPase [45]. The v-ATPase-Ragulator complex serves as an endosomal docking site for liver kinase B1 (LKB1)-mediated AMPK activation by forming the v-ATPase-Ragulator-axis inhibition protein 1 (AXIN)/LKB1-AMPK complex and thus providing a switch between catabolism and anabolism [45]. A glucose deficiency sensed by aldolase promotes the interaction of the AXIN-LKB1 complex with v-ATPase and the Ragulator complex, resulting in the activation of AMPK signaling [46, 47]. Concurrently, AXIN inhibits GEF activity toward RAG, resulting in dissociation from the endosome and the inactivation of mTORC1 by interfering with the interaction between RAGs and the Ragulator complex. The Ragulator complex is also involved in fructose-1,6-bisphosphate (FBP)-mediated glucose sensing by AMPK [46]. When unoccupied by FBP, aldolases promote the formation of a lysosomal complex containing, at minimum, v-ATPase, the Ragulator complex, axin, LKB1, and AMPK (Fig. 2).

The association between LAMTOR1 and CDKN1B/p27^Kip1^ (p27) is also well-studied [48–50]. p27 is a tumor suppressor and binds to and inhibits cyclin-CDK complexes in the nucleus, inducing cell cycle arrest [51]. During prolonged starvation, a fraction of p27 is recruited to lysosomes and interacts with LAMTOR1. Interactions between p27 and LAMTOR1 prevent Ragulator complex assembly and mTORC1 activation [48]. This interaction regulates starvation-induced cell death [49]. In LAMTOR1-deficient MEF cells, the suppression of p27 expression mitigated starvation-induced cell death by stimulating autophagy [49]. *TMEM127*, a tumor suppressor gene, interacts with the Ragulator complex in an amino acid-dependent manner and decreases the LAMTOR1-vATPase association. This interaction between TMEM127 and the Ragulator-vATPase complex at the lysosome inhibits mTORC1 signaling in response to amino acids [52]. Neurofibromin, a tumor suppressor, forms a protein complex with LAMTOR1 and inhibits mTOR signaling [53]. Zan et al. identified a feedback loop between the Ragulator complex and folliculin that regulates the mTORC1 pathway based on a quantitative genetic screen in the HAP1 haploid human cell line [54]. In this report, LAMTOR4 had a synthetic sick/lethal interaction with folliculin, and interestingly, the requirements for LAMTOR4 and LAMTOR5 in the regulation of the mTORC1 pathway differed under fed and starved conditions [54].

The Ragulator complex also regulates the position and size of lysosomes, which are important parameters for metabolism. The Ragulator complex controls lysosome positioning by direct interactions with BLOC-1-related complex (BORC), which promotes lysosome dispersal by coupling to the small GTPase Arl8 and the kinesins KIF1B and KIF5B [55, 56]. This interaction between the Ragulator complex and BORC also regulates the size of the late endosome/lysosome via PIKfyve-dependent phosphatidylinositol-3,5-bisphosphate [PI(3,5)P2] production [57]. Another analysis using Lamtor2 knockout (KO) cells showed that the Ragulator complex also contributes to the proper formation of multivesicular body-recycling tubules and the regulation of membrane/cargo recycling from multivesicular bodies [58].

Few reports have shown that the Ragulator complex contributes to metabolism in vivo. Macrophage-specific LAMTOR1 KO mice showed resistance to high-fat diet-induced obesity, lipid steatosis, and glucose metabolic disorders, with elevated levels of pro-inflammatory cytokines [59]. The loss of LAMTOR1 in pancreatic *β* cells increases glucose‑stimulated insulin secretion in mice [60].

## Multiple roles of the Ragulator complex in cellular phenomena

Although the Ragulator complex has a clearly established role as a regulator of cellular metabolic states by coordinating mTORC1 and AMPK activities, recent research has focused on its other functions (Table 1). The Ragulator complex is a platform for maintaining cellular homeostasis, with roles in integrin signaling via Lamtor2–MEK, acidification of lysosomes via V-type ATPase, lysosome biogenesis by enhanced TFEB nuclear translocation, endomembrane damage repair or organelle homeostasis, and the regulation of migration through interactions with the myosin phosphatase Rho-interacting protein (MPRIP) [5–7, 17, 18, 61]. In addition to these regulatory effects on cellular functions, a variety of studies have revealed that the Ragulator complex is tightly involved in the immune response, including the magnitude of the response, and serves as a two-way switch in the regulation of inflammation.

**Table 1:** Summary of various functions of the Ragulator complex

**Function**	**Regulation (target)**	**Mechanism (elucidated effector)**	**Ref**
Inflammatory regulation	M1/M2 polarization	LXR, TLR2	[62–64]
	Inflammatory cytokine	TFEB, TLR4	[65, 66]
	Type 1 IFN	DDX19-TBK1-IKKε complex (interaction)	[67]
	Inflammasome activation	NLRP3 and HDAC6 (interaction)	[68]
Immune cell homeostasis	Treg	SREBP	[69]
	B cell	(NA)	[70, 71]
	Langerhans cells	TGFβ receptor II	[72, 73]
Anti-tumor immunity	Antigen presentation	MHC-II	[74]
Bacterial clearance	Endo-lysosomal system	(NA)	[75–77]
Cell death	Pyroptosis	RIPK1 (interaction), ROS production	[78, 79]
	Apoptosis	ROS production	[80]
Cell migration		MPRIP (interaction)	[18, 81, 82]
CNS homeostasis	Synaptic plasticity	TRPML1 (interaction)	[83, 84]

The functions of the Ragulator complex with mechanisms to target the regulation. This list does not describe all the functions and mechanisms involving direct mTORC1 regulation. The included functions are selected as representative examples based on reports, and there may be overlaps between them (*NA* not applicable)

### Cytokine production

Lamtor1 is critical for the M2 polarization of macrophages [62]. Lamtor1-deficient macrophages do not express M2 signature genes after stimulation with IL-4. Mechanistically, Lamtor1 controls the expression levels of M2 signature genes by promoting the production of oxysterol, which activates the transcription factor LXR [62]. Another group has identified the importance of the regulation of M2 macrophage polarization via Lamtor1 in wound healing [63]. They showed that acellular dermal matrix scaffolds generate a pro-regenerative microenvironment during full-thickness cutaneous wound healing via M2 macrophage polarization. Additionally, Davis et al. reported the role of Lamtor1 in the phagocytosis-mediated M1 activation of macrophages by chitin with Toll-like receptor 2 [64]. Lamberti et al. have shown that LAMTOR/Ragulator regulates lipid metabolism in macrophages and foam cell differentiation [85]. In the case of macrophages, loss of Lamtor1 enhanced the innate immune response by accelerating the nuclear translocation of TFEB [65]. LAMTOR1 regulates the NOD2- and TLR2-mediated phosphorylation of the deubiquitinase ataxin-3, which plays important roles in innate immune sensing and metabolism in myeloid cells [86]. Lamtor5 regulates inflammation by regulating the autophagic degradation of TLR4 [66]. Zhang et al. reported that Lamtor5 is associated with TLR4, with colocalization at autolysosomes, preventing lysosomal tethering and derepressing TFEB to promote the autophagic degradation of TLR4. Therefore, Lamtor5 deficiency delays the degradation of TLR4, leading to sustained inflammation [66]. The DExD/H-box helicase family member DExD/H-box RNA helicase 19 (DDX19) negatively regulates type 1 IFN production by promoting TBK1 and IKKε degradation via the formation of a complex with LAMTOR2 [67].

### Inflammasome regulation

Recently, we reported that the Ragulator complex regulates NLRP3 inflammasome activation via interactions with HDAC6 [68]. A Lamtor1 deficiency abrogated NLRP3 inflammasome activation in murine macrophages and human monocytic cells. Myeloid-specific Lamtor1-deficient mice showed the marked attenuation of the severity of NLRP3-associated inflammatory diseases, including LPS-induced sepsis, alum-induced peritonitis, and monosodium urate-induced arthritis. Mechanistically, Lamtor1 interacts with both NLRP3 and HDAC6, and the absence of HDAC6 attenuates the interaction between Lamtor1 and NLRP3, suggesting that the Ragulator complex on lysosomes may serve as a scaffold that allows NLRP3 transported by HDAC6 to increase locally [68].

### Immune cell homeostasis

The loss of Lamtor1 is critical for the suppressive function of regulatory T cells [69]. Foxp3-Cre-driven conditional Lamtor1 KO mice develop systemic autoimmunity and die within 1 month of birth. Lamtor1 promotes the suppressive function of regulatory T cells by activating the expression of SREBP target genes [69]. LAMTOR2 was initially reported to confine MAPK signaling to late endosomes [36, 37]. Additional research has focused on the roles of Lamtor2 in patients with primary immune deficiency syndrome with endosomal/lysosomal defects in immune cells suffering from recurrent bronchopulmonary infections [70]. Patients with LAMTOR2 point mutations showed reduced B-cell maturation, class switching, and memory cell formation as well as the impaired ability of neutrophils to kill bacteria, without affecting phagocytosis [70]. Subsequent research indicated that LAMTOR2 is critical for the generation and activation of mature B lymphocytes [71]. Delayed receptor internalization and endosomal trafficking due to a LAMTOR2 deficiency resulted in aberrant BCR signaling; therefore, the conditional deletion of LAMTOR2 at the pre-B1 stage using mb1-Cre mice resulted in complete developmental arrest [71]. Gene co-expression network analysis identified the involvement of Lamtor2 in platelet regulation in COVID-19 [87].

### Antimicrobial functions

Lamtor1 was first identified as an essential protein for anchoring the MEK-ERK pathway to late endosomes and for controlling endosome dynamics [61]. Shi et al. reported that SLC38A9, v-ATPase, and the Ragulator complex (but not Rag GTPases and mTORC1) are essential for amino acid-stimulated endosome-to-Golgi trafficking [88]. In this process, the small GTPase Arl5 interacts with the Ragulator complex in an amino acid-dependent manner. The regulation of membrane trafficking by the Ragulator complex is not limited to metabolic regulation but is also involved in inflammatory and infectious disease responses. By the regulation of the endocytic pathway, LAMTOR1 decreases the expression of MHC-II on cell surfaces, resulting in reduced antigen expression in anti-tumor immunity [74]. During *Salmonella* infection, the complex of LAMTOR2 and LAMTOR3 is required for the accurately timed transport of *Salmonella* through the endolysosomal system [75]. Another group reported that LAMTOR1 and LAMTOR2 regulate xenophagy, selective antibacterial autophagy, by recruiting the autophagy receptor TAX1BP1 in response to GAS and *Salmonella* invasion [76]. LAMTOR1 was localized to bacterium-containing endosomes, and LAMTOR2 was recruited to damaged bacterium-containing endosomes in a LAMTOR1-dependent manner, facilitating autolysosome formation during bacterial infection [76]. Short-chain fatty acids activate G protein-coupled receptor 43 (GPCR43) to increase macrophage bacterial clearance by upregulating LAMTOR2 [77].

## Cell death

Lysosomes mediate cell death at several levels. The Ragulator complex also regulates cell death in various manners, and this function has been a major focus of research [89–91]. A series of recent studies have revealed the relationship between the Ragulator complex and pyroptosis, a type of inflammatory caspase-1-dependent cell death [78, 79]. In pyroptosis, the Ragulator complex plays an essential role in the regulation of Gasdermin D (GSDMD) oligomerization and pore formation [78, 79]. Devant et al. also reported that the Ragulator complex is required for reactive oxygen species production, which enables the oligomerization and pore formation of GSDMD. The regulation of reactive oxygen species production by LAMTOR1 regulates p53-dependent cell cycle arrest and apoptosis independently of mTORC1 activity [80]. LAMTOR2-deficient animals displayed the increased apoptosis of Langerhans cells, resulting in the nearly complete loss of the Langerhans cell network in the epidermis [72]. Subsequent analysis showed that a LAMTOR2 deficiency in dendritic cells or Langerhans cells interferes with the transforming growth factor β1 (TGFβ1) pathway, by lowering TGFβ receptor 2 expression, which is essential for the homeostasis of Langerhans cells [73]. The relationship between *N*-myristoyltransferase-1 (NMT1), which functions as an oncogene in various cancers, and Lamtor1 has been emphasized in cancer biology by several groups [92]. NMT1 myristoylates LAMTOR1 at Gly 2 resulting in increased LAMTOR1 protein stability and lysosomal localization. This modification is correlated with bladder cancer progression [93].

The total knockout of each Ragulator complex leads to embryonic lethality [61, 94]. Recently, Qin et al. reported that the knockout of LAMTOR5, a well-characterized transcriptional coactivator in various cancers, leads to embryonic lethality, with retarded growth around embryonic day 7.5. The depletion of LAMTOR5 compromises the self-renewal of embryonic stem cells, with reduced expression of pluripotency genes, reduced cell proliferation, and decreased colony-forming capacity [94].

## Cell migration

Several studies have demonstrated that various components of the Ragulator complex are involved in cell migration, an important process in inflammation in various mouse models. Early studies of Lamtor1 revealed its relationship to cell migration [61]. We have recently reported that the Ragulator complex plays an essential role in leukocyte trafficking by activating myosin II via interactions with MPRIP [18]. In brief, during cell migration, lysosomes move to the uropod, and Lamtor1 on the lysosomes interacts with MPRIP and interferes with the interaction between MPRIP and MYPT1, a subunit of myosin light chain phosphatase (MLCP). This interaction results in increasing myosin II-mediated actomyosin contraction [18]. Consistent with this Lamtor1 KO phenotype, mice with LAMTOR2-deficient dendritic cells have a severe disturbance of the dendritic cell compartment [81]. Another group has shown that the LAMTOR2/3 scaffold complex was transported to the vicinity of focal adhesions and regulates cell migration [82]. The regulation of these migratory activities by the Ragulator complex contributes to the induction of inflammation in various mouse models [18].

## Maintenance of homeostasis in the central nervous system

Abnormal mTORC1 activation has been implicated in several developmental neurological disorders, and during neuronal development, mTORC1 responds to glutamate and neurotrophins to promote neuronal migration and dendritic arborization [95]. The role of the Ragulator complex in the central nervous system is also well-reported. Recent transcriptional profiling of the olfactory pathway of female African green monkeys, a well-described model of early Alzheimer’s disease-like neuropathology, has suggested that LAMTOR3 is involved in Alzheimer’s disease phenotypes [96]. Genome-wide association studies have identified that Lamtor4 is a prognostic indicator for Alzheimer’s disease [97]. A gene co-expression network approach using data from three patients with Alzheimer’s disease suggested that LAMTOR1 is a candidate biomarker [98]. Additionally, the expression of LAMTOR4 is significantly lower in the blood of patients with schizophrenia than in a control group [99]. Lamtor4 is an essential regulator of lysosomes in microglia in zebrafish [100]. Ube3a-mediated regulation of LAMTOR1 is critical for typical synaptic plasticity, dendritic spine development, and learning and memory [83]. Sun et al. reported that in a ubiquitin E3 ligase, UBE3A ubiquitinates LAMTOR1, resulting in its proteasomal degradation, and Lamtor1 knockdown in hippocampal CA1 neurons of mice with Angelman syndrome reduces elevated mTORC1 activity and improves dendritic spine maturation, long-term potentiation, as well as learning performance [83]. LAMTOR1-mediated inhibition of TRPML1-dependent lysosomal calcium release regulates dendritic lysosome trafficking and hippocampal neuronal function [84]. The Ragulator complex restricts lysosomal trafficking in dendrites of hippocampal neurons via TRPML1 activity through the interaction between Lamtor1 and TRPML1. The Ragulator-mediated inhibition of TRPML1 is critical for the regulation of dendritic lysosomal motility, synaptic plasticity, and learning. LAMTOR1 deletion in the hippocampal CA1 region of adult mice results in alterations in synaptic plasticity and in impaired object-recognition memory and contextual fear conditioning due to TRPML1 activation [84]. In hippocampal neurons, LAMTOR1 is dynamically palmitoylated prior to mTORC1 activation, and this modification is important for mTORC1 signaling [95]. There is evidence for the involvement of the interaction between LAMTOR1 and p27 in membrane trafficking and microtubule-dependent transport in postmitotic cortical neurons [50]. Ratnayake et al. reported that uv90, homologous to human LAMTOR1, plays a role in circadian rhythmicity in *Neurospora crassa* [101].

## Conclusions

Lysosomes serve as a signaling hub, integrating extracellular and intracellular stimuli. In addition to the regulation of metabolism, the Ragulator complex also functions in the coordination of inflammation, cell death, migration, and the maintenance of homeostasis. This multi-potency can be explained by its role, a platform for integrating cellular information via interactions with various proteins. The Ragulator complex is thought to possess numerous as-yet-unreported interactions with various molecules. Further examination of these interactions has the potential to not only regulate metabolism via mTORC1 but also understand the management of various physiological processes and pathological conditions.

## Data Availability

Not applicable.

## Abbreviations

AMPK: AMP-activated protein kinase
AXIN: Axis inhibition protein 1
BORC: BLOC-1-related complex
ERK: Extracellular-signal-regulated kinase
FBP: Fructose-1,6-bisphosphate
GEF: Guanine nucleotide exchange factor
GSDMD: Gasdermin D
GTP: Guanosine triphosphate
KO: Knockout
LKB1: Liver kinase B1
MAPK: Mitogen-activated protein kinase
MLCP: Myosin light chain phosphatase
MPRIP: Myosin phosphatase Rho-interacting protein
mTORC1: Mammalian target of rapamycin complex 1
NMT1: N-Myristoyltransferase-1
PI3K: Phosphatidylinositol-3-kinase
RAG: RAS-related GTP-binding proteins
TGFβ1: Transforming growth factor β1
